# Median effective dose of fospropofol disodium for suppression of the cough reflex during tracheostomy tube exchange in patients with disorders of consciousness receiving fixed-dose sufentanil

**DOI:** 10.3389/fphar.2026.1909624

**Published:** 2026-09-10

**Authors:** Chaoyang Chen, Lijun Wu, Xinxin Yue, Qing Cheng, Xiang Li, Zeyu Zhao

**Affiliations:** Department of Anesthesiology, Sichuan Provincial Rehabilitation Hospital, Chengdu, Sichuan, China

**Keywords:** cough response, disorders of consciousness, fospropofol disodium, median effective dose, sequential method, tracheostomy

## Abstract

**Objective:**

The aim of this study was to determine the median effective dose (ED_50_) and 95% effective dose (ED_95_) of fospropofol disodium required to suppress the cough response during tracheostomy tube exchange in patients with disorders of consciousness receiving a background dose of sufentanil 5 μg/kg.

**Methods:**

Twenty-nine patients with disorders of consciousness requiring general anesthesia for surgical procedures were enrolled. All had a pre-existing tracheostomy and were classified as ASA IV. General anesthesia was induced with intravenous sufentanil 5 μg/kg, followed by administration of fospropofol disodium using a sequential allocation design. A cough response graded as III or higher was defined as a positive response. The study was terminated after the occurrence of 10 effective crossover points. Probit regression analysis was used to estimate the ED_50_, ED_95_, and corresponding 95% confidence intervals. Mean arterial pressure (MAP), heart rate (HR), and respiratory rate (RR) was recorded before anesthesia induction (T0), before tracheostomy tube exchange (T1), and immediately after tube exchange (T2). The incidences of hypotension and apnea were also recorded.

**Results:**

The ED_50_ and ED_95_ of fospropofol disodium for suppression of the cough response were 9.41 mg/kg (95% CI, 8.08–10.64 mg/kg) and 12.66 mg/kg (95% CI, 11.01–26.05 mg/kg), respectively. Repeated-measures analysis of variance demonstrated significant time-dependent effects on MAP and RR (both p < 0.001), whereas HR demonstrated no significant changes over time (p > 0.05). Compared with T0, MAP and RR were significantly reduced at T1 and T2 (both p < 0.01), with no statistically significant differences observed between T1 and T2 (both p > 0.05). The incidence rates of hypotension, apnea, and oxygen desaturation were higher in the 12 mg/kg group than in the 10 mg/kg group (66.67% vs. 15.38%, 50.00% vs. 7.69%, and 16.67% vs. 0%, respectively).

**Conclusion:**

In the presence of sufentanil 5 μg/kg, the ED_50_ and ED_95_ of fospropofol disodium for suppressing the cough reflex during tracheostomy tube exchange in patients with disorders of consciousness were 9.41 mg/kg (95% CI, 8.08–10.64 mg/kg) and 12.66 mg/kg (95% CI, 11.01–26.05 mg/kg), respectively. The suppressive effect was dose-dependent; however, higher doses were associated with increased risks of hypotension and apnea, indicating the need for individualized dose titration.

## 1 Introduction

Tracheostomy is commonly performed to maintain long-term airway patency in patients with disorders of consciousness (Dickey et al., 2026). Prior to induction of general anesthesia, the preexisting tracheostomy tube is typically replaced with an anesthesia-compatible tube. Although consciousness is impaired in this patient population, the brainstem-mediated cough reflex is often preserved (Al-Biltagi et al., 2022). Without patient cooperation, airway stimulation during tube exchange may readily provoke coughing, potentially leading to abrupt increases in blood pressure, elevated intracranial pressure, airway injury, and interference with subsequent anesthesia induction (Wang et al., 2025; Chen et al., 2020). Fospropofol disodium, a novel water-soluble prodrug of propofol, has been demonstrated to effectively suppress airway reflexes (Zou et al., 2025). Sufentanil, a potent μ-opioid receptor agonist, also attenuates airway reflex activity and suppresses the cough response (Ji et al., 2016). However, the optimal dose of fospropofol disodium required to suppress coughing during tracheostomy tube exchange in patients with disorders of consciousness receiving sufentanil has not yet been established. Therefore, this study was conducted to determine the median effective dose (ED_50_) of fospropofol disodium administered along with a fixed dose of sufentanil for suppression of the cough response during tracheostomy tube exchange prior to induction of anesthesia in patients with disorders of consciousness. The 95% effective dose (ED_95_) was also estimated to provide a reference for clinical anesthetic dosing.

## 2 Materials and methods

### 2.1 General data

Patients with disorders of consciousness who were scheduled to undergo surgery at the Sichuan Provincial Bayi Rehabilitation Center between April 2025 and March 2026 were enrolled in this study.

The inclusion criteria were as follows: (1) age between18 to 64 years, irrespective of sex; (2) diagnosis of unresponsive wakefulness syndrome or minimally conscious state; (3) presence of a tracheostomy; (4) American Society of Anesthesiologists Physical Status IV; (5) modified frailty index score ≥3; and (6) planned surgical treatment under general anesthesia.

The exclusion criteria were as follows: (1) history of allergy to analgesic or sedative medications; (2) use of benzodiazepines within the preceding month; (3) concomitant myasthenia gravis; (4) uncontrolled stage 3 hypertension; (5) fever or uncontrolled pulmonary infection; and (6) preoperative alkaline phosphatase levels below the lower limit of normal (45 U/L). The elimination criteria included: (1) unplanned cancellation of surgery after induction of anesthesia; and (2) failure of tracheostomy tube exchange on the first attempt. The study was registered with the Chinese Clinical Trial Registry (ChiCTR; Registration No. ChiCTR2500096769) and approved by the Institutional Ethics Committee (Approval No. CKLL-20241214). Per the registry requirements, a Data and Safety Monitoring Committee (DSMC) consisting of three independent experts (one clinician, one anesthesiologist, and one medical biostatistician) was established prior to trial initiation to oversee the periodic review of safety data during the trial. Written informed consent was obtained from the family members or legal representatives of all participants.

### 2.2 Anesthesia induction and sequential administration protocol

All participants underwent fasting from food and liquids, or discontinuation of nasogastric feeding, for at least 8 h prior to surgery. Upon arrival in the operating room, standard monitoring was established, including electrocardiography, noninvasive blood pressure, pulse oxygen saturation (SpO_2_), and Bispectral Index monitoring. Penehyclidine hydrochloride 0.3 mg was administered intravenously, and oxygen was delivered via the tracheostomy tube at a flow rate of 2 L/min.

Anesthesia induction was initiated with intravenous sufentanil 5 μg/kg (Yichang Humanwell Pharmaceutical Co., Ltd.; Batch No. AB40900921). Fospropofol disodium (Yichang Humanwell Pharmaceutical Co., Ltd.; Batch No. AC5020011) was subsequently administered according to a sequential allocation design. The initial dose administered to the first participant was 10 mg/kg, with a dose interval of 2 mg/kg, corresponding to one-fifth of the initial dose. Tracheostomy tube exchange, defined as the replacement of the existing tube with an anesthesia-compatible tube, was performed 5 min after completion of fospropofol disodium administration.

Dose adjustment was conducted according to the observed response during tube exchange. In the absence of a positive response, the dose administered to the subsequent participant was decreased by 2 mg/kg. When a positive response occurred, the dose for the subsequent participant increased by 2 mg/kg.

The cough response during tube exchange was graded as follows: Grade I, no cough; Grade II, mild cough (<3 episodes); Grade III, ≥3 cough episodes lasting ≤5 s; and Grade IV, persistent coughing lasting >5 s (Tung et al., 2019). This grading system was applied uniformly by a single trained anesthesiologist, and the assessment was performed immediately after tube exchange. A cough response of Grade III or higher was defined as a positive response (unsuccessful suppression). All cough responses were evaluated by an anesthesiologist who was blinded to dose allocation and the sequential allocation sequence, ensuring that the assessment of the primary endpoint was not influenced by knowledge of the assigned dose.

Dose estimation was performed using the truncated Dixon–Mood method combined with regression analysis (Oron et al., 2022). In accordance with statistical precision requirements, the study was terminated when 10 effective crossover points had been observed, as this stopping rule is consistent with methodological recommendations for sequential dose-finding designs, in which 20–40 observations are generally considered sufficient for stable ED_50_ estimation (Tan et al., 2022). The number of participants enrolled at study termination was defined as the final sample size.

### 2.3 Outcome measures

Mean arterial pressure (MAP), heart rate (HR), respiratory rate (RR), and SpO_2_ were recorded at the following time points: prior to the induction of anesthesia (T0), prior to the tracheostomy tube exchange (T1), and immediately after tube exchange (T2). The incidences of cough response, hypotension, and apnea were also documented. Hypotension was defined as a reduction in MAP greater than 20% from baseline. Apnea was defined as cessation of breathing for >30 s or the need for manual ventilation support. Oxygen desaturation was defined as SpO_2_ < 90% requiring intervention, such as jaw thrust, increased oxygen flow, or bag-mask ventilation. Cough incidence was calculated as follows: (number of patients with cough response/total number of patients) × 100%.

### 2.4 Statistical analysis

Statistical analyses were performed using SPSS version 31.0. Normally distributed continuous variables were expressed as mean ± standard deviation (x̄ ± s). Variables measured repeatedly at different time points were analyzed using repeated-measures analysis of variance (ANOVA), followed by Bonferroni *post hoc* correction. A two-sided p < 0.05 was considered statistically significant.

The up-and-down sequential allocation method was used to determine the dose range and to derive a preliminary estimate of the ED_50_. However, this design is not inherently optimized for estimating high-percentile effective doses such as the ED_95_. To further estimate the ED_50_, ED_95_, and their corresponding 95% CIs, we employed probit regression analysis based on the full dose–response data across all dose levels. Under this model, the probability of a positive cough response was modeled as a function of the logarithm of dose, with parameters estimated by maximum likelihood. The 95% CIs were calculated using the profile likelihood method. It should be noted that this probit-based extrapolation to the ED_95_ is exploratory in nature, given the dose concentration near the ED_50_ inherent to the up-and-down design, and the results should be interpreted with caution. All probit regression analyses were performed using SPSS version 31.0 (IBM Corp., Armonk, NY, USA), and dose–response curves were generated using GraphPad Prism version 11.0 (GraphPad Software, Boston, MA, USA).

## 3 Results

### 3.1 Patient general characteristics

A total of 29 patients were enrolled, yielding 10 effective crossover points. The study population comprised of 19 males and 10 females, with an age range of 21–64 years and a mean age of 52.59 ± 11.33 years. The mean body mass index was 22.01 ± 1.98 kg/m^2^. The surgical procedures included cranioplasty in 18 patients, spinal cord stimulator implantation in 7 patients, ventriculoperitoneal shunt placement in 3 patients, and cerebrospinal fluid leak repair in 1 patient. Detailed data are presented in Table 1.

**TABLE 1:** Baseline characteristics of patients (N = 29).

Characteristics	Value
Age (years, mean ± SD)	52.59 ± 11.33
Sex (n, %)	
Male	19 (65.52)
Female	10 (34.48)
BMI (kg/m^2^, mean ± SD)	22.01 ± 1.98
Etiology (n, %)	
Traumatic brain injury	17 (58.62)
Hemorrhagic stroke	12 (41.38)
Diagnosis (n, %)	
UWS	7 (24.14)
MCS	22 (75.86)
Tracheostomy duration (months, median [IQR])	11.0 (7.5–14.5)
mFI score (points, mean ± SD)	3.24 ± 0.51
Surgical procedure (n, %)	
Cranioplasty	18 (62.07)
Spinal cord stimulator implantation	7 (24.14)
Ventriculoperitoneal shunt	3 (10.34)
CSF leak repair	1 (3.45)
Comorbidities	
Hypertension (n, %)	6 (20.69)
Coronary artery disease (n, %)	5 (17.24)
Diabetes mellitus (n, %)	3 (10.34)

BMI, body mass index; mFI, modified frailty index; UWS, unresponsive wakefulness syndrome; MCS, minimally conscious state; CSF, cerebrospinal fluid.

### 3.2 Implementation of the sequential method and determination of ED_50_ and ED_95_

**FIGURE 1:**
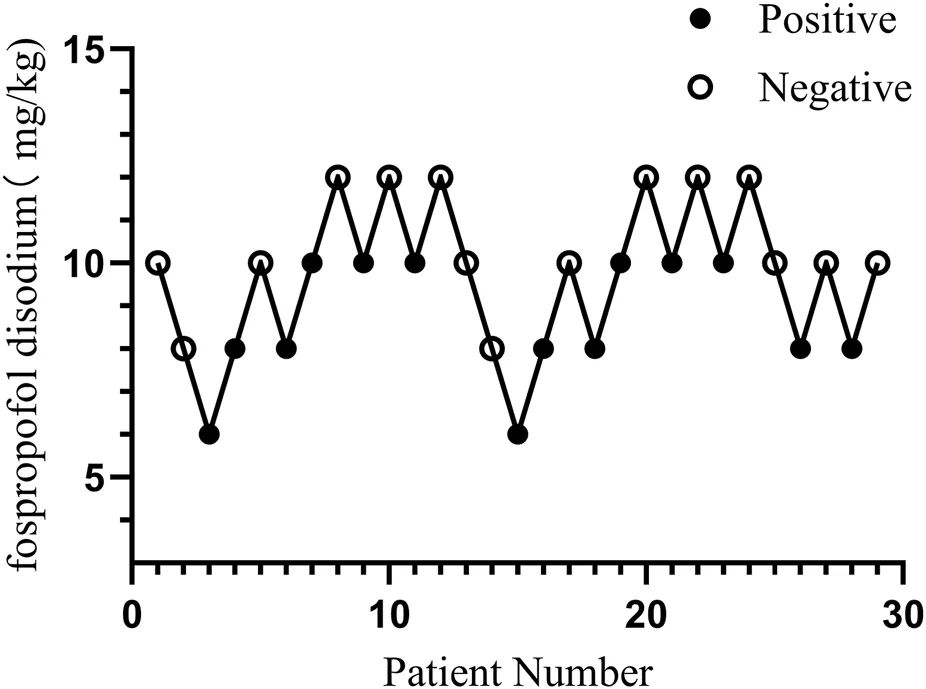
Dose-sequence plot of fospropofol disodium for suppressing cough response during tracheal tube exchange.

**FIGURE 2:**
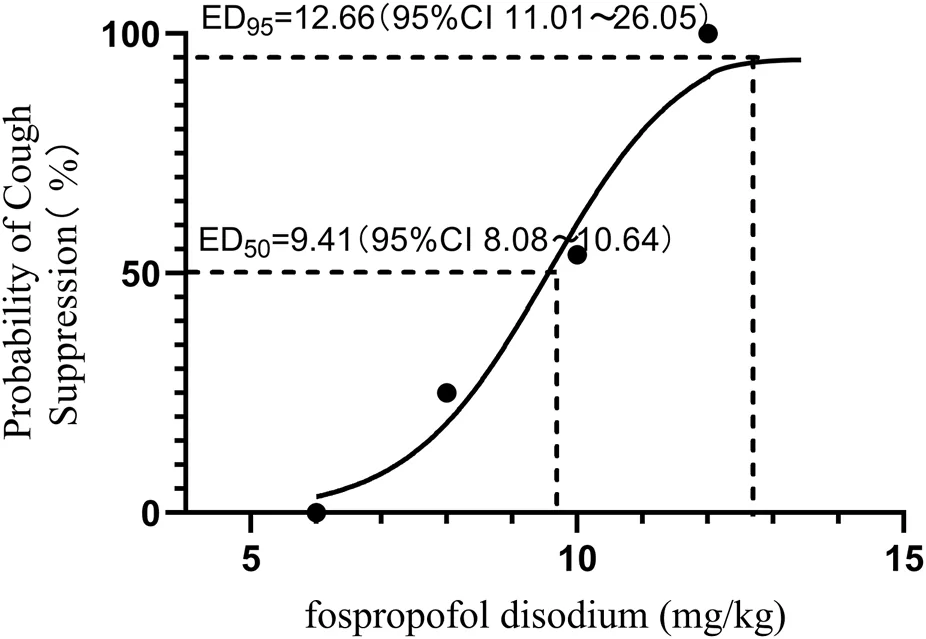
Dose-effect curve of fospropofol disodium for suppressing cough response during tracheal tube exchange.

The sequential allocation method was used to estimate the ED_50_ of fospropofol disodium for suppression of the cough response during tracheostomy tube exchange. A dose-dependent effect was observed, with the incidence of coughing progressively decreasing as the dose of fospropofol disodium increased. According to Dixon–Mood analysis, the ED_50_ of fospropofol disodium for suppression of the cough response was 9.41 mg/kg (95% CI, 8.08–10.64 mg/kg). The estimated ED_95_ was 12.66 mg/kg (95% CI, 11.01–26.05 mg/kg). Detailed results are presented in Table 2 and Figures 1, 2.

**TABLE 2:** Sequential allocation analysis of cough responses across different fospropofol disodium dose groups.

Fospropofol disodium dose (mg/kg)	Total number of patients (*n*)	Number of coughing cases (*n*)	Incidence of cough (%)
6	2	2	100.00
8	8	6	75.00
10^*^	13	6	46.15
12	6	0	0.00

*Initial dose.

### 3.3 Changes in MAP, HR, and RR

Mauchly’s test of sphericity demonstrated that the assumption of sphericity was satisfied for all variables (all p > 0.05); therefore, the original degrees of freedom were retained for repeated-measures ANOVA. Significant time-dependent effects were observed for both MAP and RR (both p < 0.001), whereas no significant time effect was identified for HR (p > 0.05). Bonferroni *post hoc* analysis demonstrated that MAP and RR were significantly lower at T1 and T2 than at T0 (both p < 0.01). However, no statistically significant differences in MAP or RR were observed between T1 and T2 (both p > 0.05). HR did not differ significantly among the three time points (all p > 0.05). Detailed data are presented in Table 3. Individual MAP values at T0, T1, and T2 for each patient are provided in Supplementary Table S1.

**TABLE 3:** Comparison of MAP, HR, and RR at different time points (*x̄̅* ± *s*).

Variables	T0	T1	T2	F (df)	P	ηp^2^	Post-hoc test
MAP (mmHg)	98.3 ± 10.6	83.4 ± 15.2	81.2 ± 14.9	23.309 (2,56)	<0.001	0.454	T0>T1, T2*
HR (bpm)	79.6 ± 17.3	80.3 ± 20.8	82.4 ± 17.5	0.455 (2,56)	0.636	0.016	T0vsT1, T2, n.s
RR (breaths/min)	19.0 ± 0.3	12.8 ± 1.1	9.9 ± 1.4	29.420 (2,56)	<0.001	0.634	T0>T1, T2*

*p < 0.01; n.s.: no significance. The above sphericity tests satisfied *p* > 0.05, and the original degrees of freedom were used.

### 3.4 Incidence of adverse events

In the fospropofol disodium 10 mg/kg group (n = 13), the incidences of hypotension, apnea, and oxygen desaturation were 15.38% (2/13), 7.69% (1/13), and 0% (0/13), respectively. In the 12 mg/kg group (n = 6), the incidences of hypotension, apnea, and oxygen desaturation were 66.67% (4/6), 50.00% (3/6), and 16.67% (1/6), respectively.

## 4 Discussion

This study demonstrated that, in patients with disorders of consciousness receiving a background dose of sufentanil 5 μg/kg, the ED_50_ of fospropofol disodium required to suppress the cough response during tracheostomy tube exchange was 9.41 mg/kg. The cough reflex is mediated through a complex reflex arc involving tracheal mucosal receptors, afferent and efferent vagal pathways, and medullary centers, including the nucleus tractus solitarius and nucleus ambiguus (Mazzone and Farrell, 2019). Receptor density is most concentrated at the carina, and stimulation of this region may trigger a carinal reflex cough that is substantially more intense than coughing induced by routine airway stimulation (Taylor-Clark, 2021). In this study, the insertion depth of the tracheal tube was strictly maintained at 6–8 cm from the tracheostomy stoma to avoid contact between the catheter tip and the carina. This strategy minimized interference from the carinal reflex and enabled more accurate estimation of the ED_50_.

The mechanism by which fospropofol disodium suppresses the cough response may be explained as follows. Fospropofol disodium is a pharmacologically inactive prodrug that undergoes hydrolysis by alkaline phosphatase after intravenous administration, releasing the active metabolite propofol (Gao et al., 2024). Propofol binds to the β-subunit of γ-aminobutyric acid type A (GABA_A) receptors, enhances chloride ion (Cl^−^) channel opening, and promotes Cl^−^ influx. These effects generate inhibitory postsynaptic potentials and induce hyperpolarization of neurons within the medullary nucleus tractus solitarius and nucleus ambiguus. Consequently, excitability of the cough center is reduced, vagally mediated efferent transmission is attenuated, and suppression of the cough reflex is achieved (Wu et al., 2021). This inhibitory effect primarily involves the central efferent pathway rather than blockade of peripheral receptors or afferent nerves, thereby allowing partial preservation of airway protective reflexes. After adjustment for dose equivalence, the ED_50_ identified in this study (9.41 mg/kg) was comparable to the reported ED_50_ of propofol for suppression of coughing during tracheal intubation, which ranges from approximately 1.5–2.5 mg/kg (Zhang et al., 2025; Kwak et al., 2014). This finding further supports the pharmacodynamic consistency of fospropofol disodium as a propofol prodrug.

In comparison with remimazolam, an ultrashort-acting benzodiazepine sedative recently introduced for procedural sedation, fospropofol disodium appears to offer a more favorable profile for suppression of airway reflexes due to its GABA_A receptor-mediated mechanism, although remimazolam may provide superior hemodynamic stability in certain settings (Zhang et al., 2024). However, direct head-to-head comparisons of these agents for cough suppression during tracheostomy tube exchange are currently lacking, and the dose-response parameters established in this study provide a reference for future comparative trials. Recent evidence on fospropofol in airway procedures supports our findings. A 2026 trial comparing fospropofol (12.5 mg/kg) with propofol (2 mg/kg) for EBUS-TBNA reported more stable hemodynamics with less hypotension, but noted dose-related paresthesia and prolonged induction (Hu et al., 2026). For bronchoscopy, fospropofol 6.5 mg/kg achieved 88.7% sedation success with transient paresthesia (Silvestri et al., 2009). Compared with remimazolam (cough incidence reduced from 76.47% to 38.24% in thyroid surgery) (Liu et al., 2026), fospropofol offers a distinct GABA_A-mediated central mechanism but with different risk profiles. These comparisons reinforce that optimal sedation for airway procedures remains context-dependent, and our dose estimates provide a useful reference for future comparative studies.

Regarding hemodynamic parameters, MAP was significantly reduced at T1 and T2 compared with baseline (T0). The reduction observed at T1 indicates that fospropofol disodium may exert vasodilatory effects, whereas the absence of a significant difference between T1 and T2 indicates that tracheostomy tube exchange did not induce a compensatory increase in blood pressure (BP). Although coughing may intrinsically produce transient elevations in BP, suppression of the cough response by fospropofol disodium appeared to attenuate this hypertensive response. This observation is consistent with previous studies demonstrating that propofol suppresses cardiovascular responses associated with tracheal intubation (Zhang et al., 2025). No significant differences in HR were observed across the assessed time points, indicating that fospropofol disodium exerts minimal effects on heart rate, potentially because of limited interference with cardiac autonomic regulation (Pereira et al., 2024). This hemodynamic profile may be advantageous in patients with coexisting coronary artery disease or susceptibility to cardiac arrhythmias.

Regarding respiratory effects, RR was significantly reduced at T1 and T2 compared with baseline (T0), indicating respiratory depression associated with fospropofol disodium administration. The underlying mechanism may involve suppression of carbon dioxide responsiveness within the medullary respiratory center by active propofol generated through fospropofol disodium metabolism, thereby resulting in reductions in respiratory rate or the occurrence of apnea (Hannam et al., 2016). The incidences of hypotension and apnea demonstrated an apparent dose-dependent relationship. Hypotension occurred more frequently in the 12 mg/kg group than in the 10 mg/kg group (66.67% vs. 15.38%), and a similar trend was observed for apnea (50.00% vs. 7.69%). This finding may be attributable to higher concentrations of active propofol produced at increased fospropofol disodium doses, thereby enhancing inhibitory effects on vascular smooth muscle tone and the medullary respiratory center.

The observed dose-response relationship follows a sigmoidal pattern, which is consistent with the pharmacodynamics of propofol at GABA_A receptors (Woll et al., 2016). The steepness of the dose-response curve in the ED_50_–ED_95_ range suggests that relatively small dose increments may produce substantial incremental suppression of the cough reflex, but this comes at the cost of a proportionally greater increase in adverse events. This relationship is particularly clinically relevant in patients with disorders of consciousness, in whom the baseline cough reflex may be altered by central nervous system injury, potentially affecting the sensitivity to centrally acting sedatives.

From a clinical perspective, although the ED_50_ (9.41 mg/kg) was sufficient to suppress the cough response in 50% of patients, clinically significant coughing may still occur in nearly half of cases. In contrast, the ED_95_ (12.66 mg/kg) provided more reliable suppression of the cough response but was associated with a substantially increased absolute risk of hypotension (from 15.38% at 10 mg/kg to 66.67% at 12 mg/kg, an absolute risk increase of 51 percentage points) and apnea (from 7.69% to 23.08%). Oxygen desaturation (SpO_2_ < 90%) occurred in 16.67% of patients in the 12 mg/kg group, predominantly within the first 5 min after fospropofol disodium administration, and resolved promptly with jaw thrust or increased oxygen flow; no patient required bag-mask ventilation or reintubation. These findings indicate that while the ED_95_ dose provides more reliable cough suppression, the clinical benefit must be weighed against the markedly elevated risk of adverse events. The absolute incidence rates reported herein provide more tangible safety benchmarks for dose selection than P values alone, facilitating individualized risk-benefit assessment in clinical decision-making. Consequently, individualized risk-benefit assessment is necessary prior to clinical administration. For patients with limited cardiopulmonary reserve or elevated intracranial pressure, the risks associated with hypotension may outweigh those associated with coughing. In such cases, administration of a dose approximating the ED_50_ (approximately 9–10 mg/kg) may be considered in combination with appropriate vasoactive support. Conversely, in patients with relatively preserved cardiopulmonary function in whom suppression of coughing is the primary clinical objective, a dose approximating the ED_95_ (approximately 12–13 mg/kg) may be considered, provided that respiratory support and hemodynamic management are readily available.

However, the clinical interpretation of the ED_95_ estimate requires caution. The relatively wide 95% CI (11.01–26.05 mg/kg) reflects considerable inter-individual variability in the dose required to achieve 95% cough suppression. This is consistent with the inherent characteristics of the up-and-down method, which provides stable estimates for the median effective dose but less precise estimates for higher quantiles. Clinically, this suggests that the dose should not be escalated to the estimated ED_95_ routinely. Instead, an incremental titration strategy starting from approximately 9–10 mg/kg, with careful monitoring of both efficacy and adverse effects, may be more appropriate for individual patients.

Several limitations of this study should be acknowledged. First, the sequential allocation method inherently concentrates observations near the ED_50_ by design, resulting in an uneven distribution of participants across dose groups, with fewer patients at extreme doses. While this approach is consistent with methodological recommendations for sequential dose-finding designs and provided a relatively narrow 95% CI for the ED_50_ estimate (8.08–10.64 mg/kg), it limited the precision of the ED_95_ estimate, as reflected by its wider 95% CI (11.01–26.05 mg/kg). This is expected given that the up-and-down method is primarily designed to estimate the median effective dose. The wide confidence interval for the ED_95_ indicates that the extrapolated point estimate is associated with considerable statistical uncertainty, and thus should not be interpreted as a fixed clinical threshold. Rather, it serves as an exploratory reference that requires validation. Therefore, while this study provides a robust estimate of ED_50_, the ED_95_ estimate should be interpreted with caution, and future studies with larger sample sizes or alternative designs are needed to define the ED_95_ more precisely. Second, a fixed sufentanil dose of 5 μg/kg was used; therefore, variations in the ED_50_ of fospropofol disodium under different sufentanil dosing regimens could not be assessed. Different opioid agents or dosing strategies may alter the dose-response relationship of fospropofol disodium. Third, the study population was limited to patients with disorders of consciousness (UWS or MCS), and the relatively small sample size inherent to the sequential design precluded subgroup analyses to evaluate potential effect modification by demographic or clinical characteristics, such as age, BMI, etiology, or baseline consciousness level. Whether these factors influence the dose-response relationship of fospropofol disodium remains to be determined in larger cohorts. Therefore, extrapolation of these findings to conscious individuals or patients with different clinical characteristics should be undertaken cautiously. The impact of consciousness level on the dose-response relationship warrants further investigation. Additionally, other potential effect modifiers, including tracheostomy duration and baseline comorbidities, could not be formally assessed due to the limited sample size. Whether these factors modify the dose–response relationship remains to be determined in future larger-scale studies.

## 5 Conclusion

In patients with disorders of consciousness receiving sufentanil 5 μg/kg, the ED_50_ and ED_95_ of fospropofol disodium for suppression of cough during tracheostomy tube exchange were 9.41 mg/kg (95% CI: 8.08–10.64) and 12.66 mg/kg (95% CI: 11.01–26.05), respectively. The drug suppressed cough in a dose-dependent manner but caused dose-related reductions in MAP and RR, with higher doses associated with increased hypotension and apnea. These findings support careful dose titration, balancing efficacy against safety. Given the wide confidence interval for ED_95_, confirmation in larger, preferably multicenter studies is warranted before clinical implementation.

## Data Availability

The original contributions presented in the study are included in the article/Supplementary Material, further inquiries can be directed to the corresponding author.
