# Supplementary material for: Median effective dose of fospropofol disodium for suppression of the cough reflex during tracheostomy tube exchange in patients with disorders of consciousness receiving fixed-dose sufentanil
Source: Front Pharmacol. 2026 Sep 10;17:1909624. doi: 10.3389/fphar.2026.1909624 (PMC13601260; doi:10.3389/fphar.2026.1909624)
Supplement: Supplementary file 1 [file Table1.docx]

**Supplementary Table S1. Mean Arterial Pressure (MAP) at different time points across fospropofol disodium dose groups**

| Patient No. | Fospropofol dose (mg/kg) | MAP T0 (mmHg) | MAP T1 (mmHg) | MAP T2 (mmHg) | Cough Response |
| --- | --- | --- | --- | --- | --- |
| 1 | 10 | 95 | 90 | 81 | 0 |
| 2 | 8 | 94 | 108 | 94 | 0 |
| 3 | 6 | 108 | 120 | 94 | 1 |
| 4 | 8 | 101 | 101 | 93 | 1 |
| 5 | 10 | 101 | 87 | 67 | 0 |
| 6 | 8 | 97 | 82 | 96 | 1 |
| 7 | 10 | 95 | 105 | 93 | 1 |
| 8 | 12 | 87 | 55 | 62 | 0 |
| 9 | 10 | 90 | 70 | 112 | 1 |
| 10 | 12 | 116 | 67 | 76 | 0 |
| 11 | 10 | 88 | 65 | 64 | 1 |
| 12 | 12 | 91 | 73 | 83 | 0 |
| 13 | 10 | 101 | 79 | 89 | 0 |
| 14 | 8 | 118 | 79 | 73 | 0 |
| 15 | 6 | 85 | 79 | 69 | 1 |
| 16 | 8 | 106 | 84 | 91 | 1 |
| 17 | 10 | 90 | 76 | 76 | 0 |
| 18 | 8 | 118 | 83 | 99 | 1 |
| 19 | 10 | 112 | 93 | 89 | 1 |
| 20 | 12 | 95 | 92 | 63 | 0 |
| 21 | 10 | 107 | 105 | 69 | 1 |
| 22 | 12 | 106 | 84 | 98 | 0 |
| 23 | 10 | 91 | 80 | 61 | 1 |
| 24 | 12 | 114 | 86 | 103 | 0 |
| 25 | 10 | 91 | 61 | 72 | 0 |
| 26 | 8 | 94 | 77 | 87 | 1 |
| 27 | 10 | 83 | 68 | 60 | 0 |
| 28 | 8 | 110 | 100 | 85 | 1 |
| 29 | 10 | 85 | 71 | 58 | 0 |

**Abbreviations:** MAP, mean arterial pressure; T0, before anesthesia induction; T1, before tracheostomy tube exchange; T2, immediately after tube exchange.
